# Supplementary material for: Discovery and application of insertion-deletion (INDEL) polymorphisms for QTL mapping of early life-history traits in Atlantic salmon
Source: BMC Genomics. 2010 Mar 8;11:156. doi: 10.1186/1471-2164-11-156 (PMC2838853; doi:10.1186/1471-2164-11-156)
Supplement: Additional file 2 — Information on developed 76 locus single-run INDEL panel in Atlantic salmon. Information on fluorescence labeling, primer concentrations, PCR pooling and links to alignments, INDEL motifs and GENESCAN (Burge and Karlin 1997) predictions of genes/exons are available in html format. [file 1471-2164-11-156-S2.ZIP › Additionalfile2/table13526.html]

Contig Alignment

```
Base	A B C D E F G H   
1	G G G G G . . .    
2	G G G G G . . .    
3	G G G G G . . .    
4	G G G G G . . .    
5	T T T T T . . .    
6	T T T T T . . .    
7	A A A A A . . .    
8	A A A A A . . .    
9	A A A A A . . .    
10	A A A A A . . .    
11	T T T T T . . .    
12	A A A A A . . .    
13	G G G G G . . .    
14	T T T T T . . .    
15	T T T T T . . .    
16	T T T T T . . .    
17	T T T T T . . .    
18	T T T T T . . .    
19	A A A A A . . .    
20	T T T T T . . .    
21	A A A A A . . .    
22	A A A A A . . .    
23	A A A A A . . .    
24	A A A A A . . .    
25	A A A A A . . .    
26	C C C C C . . .    
27	T T T T T . . .    
28	G G G G G . . .    
29	A A A A A . . .    
30	A A A A A . . .    
31	C C C C C . . .    
32	A A A A A . . .    
33	T T T T T . . .    
34	G G G G G . . .    
35	C C C C C . . .    
36	T T T T T . . .    
37	T T T T T . . .    
38	C C C C C . . .    
39	T T T T T . . .    
40	C C C C C . . .    
41	T T T T T . . .    
42	A A A A A . . .    
43	G G G G G . . .    
44	G G G G G . . .    
45	C C C C C . . .    
46	T T T T T . . .    
47	T T T T T . . .    
48	T T T T T . . .    
49	C C C C C . . .    
50	A A A A A . . .    
51	C C C C C . . .    
52	A A A A A . . .    
53	C C C C C . . .    
54	T T T T T . . .    
55	T T T T T . . .    
56	A A A A A . . .    
57	G G G G G . . .    
58	T T T T T . . .    
59	A A A A A . . .    
60	T T T T T . . .    
61	T T T T T . . .    
62	A A A A A . . .    
63	C C C C C . . .    
64	A A A A A . . .    
65	A A A A A . . .    
66	T T T T T . . .    
67	A A A A A . . .    
68	A A A A A . . .    
69	T T T T T . . .    
70	A A A A A . . .    
71	T T T T T . . .    
72	T T T T T . . .    
73	A A A A A . . .    
74	C C C C C . . .    
75	T T T T T . . .    
76	C C C C C . . .    
77	T T T T T . . .    
78	C C C C C . . .    
79	T T T T T . . .    
80	T T T T T . . .    
81	C C C C C . . .    
82	T T T T T . . .    
83	C C C C C . . .    
84	A A A A A . . .    
85	T T T T T . . .    
86	T T T T T . . .    
87	C C C C C . . .    
88	A A A A A . . .    
89	G G G G G . . .    
90	T T T T T . . .    
91	A A A A A . . .    
92	C C C C C . . .    
93	A A A A A . . .    
94	A A A A A . . .    
95	T T T T T . . .    
96	A A A A A . . .    
97	C C C C C . . .    
98	A A A A A . . .    
99	A A A A A . . .    
100	A A A A A . . .    
101	C C C C C . . .    
102	C C C C C . . .    
103	A A A A A . . .    
104	G G G G G . . .    
105	T T T T T . . .    
106	T T T T T . . .    
107	T T T T T . . .    
108	G G G G G . . .    
109	A T T T T . . .    
110	G G G G G . . .    
111	A A A A A . . .    
112	G G G G G . . .    
113	A A A A A . . .    
114	T T T T T . . .    
115	G G G G G . . .    
116	A A A A A . . .    
117	A A A A A . . .    
118	T T T T T . . .    
119	G G G G G . . .    
120	A A A A A . . .    
121	A A A A A . . .    
122	G G G G G . . .    
123	C C C C C . . .    
124	A A A A A . . .    
125	A A A A A . . .    
126	A A A A A . . .    
127	C C C C C . . .    
128	A A A A A . . .    
129	G G G G G . . .    
130	C C C C C . . .    
131	C C C C C . . .    
132	A A A A A . . .    
133	A A A A A . . .    
134	A A A A A . . .    
135	T T T T T . . .    
136	A A A A A . . .    
137	A A A A A . . .    
138	A A A A A . . .    
139	T T T T T . . .    
140	A A A A A . . .    
141	C C C C C . . .    
142	T T T T T . . .    
143	G G G G G . . .    
144	T T T T T . . .    
145	G G G G G . . .    
146	C C C C C . . .    
147	T T T T T . . .    
148	A A A A A . . .    
149	A A A A A . . .    
150	T T T T T . . .    
151	G G G G G . . .    
152	T T T T T . . .    
153	C C C C C . . .    
154	A A A A A . . .    
155	C C C C C . . .    
156	T T T T T . . .    
157	T T T T T . . .    
158	A A A A A . . .    
159	G G G G G . . .    
160	T T T T T . . .    
161	A A A A A . . .    
162	T T T T T . . .    
163	A A A A A . . .    
164	C C C C C . . .    
165	T T T T T . . .    
166	A A A A A . . .    
167	A A A A A . . .    
168	T T T T T . . .    
169	G G G G G . . .    
170	T T T T T . . .    
171	A A A A A . . .    
172	A A A A A . . .    
173	A A A A A . . .    
174	T T T T T . . .    
175	G G G G G . . .    
176	A A A A A . . .    
177	T T T T T . . .    
178	A A A A A . . .    
179	A A A A A . . .    
180	T T T T T . . .    
181	A A A A A . . .    
182	T T T T T . . .    
183	T T T T T . . .    
184	G G G G G . . .    
185	C C C C C . . .    
186	C C C C C . . .    
187	C G C C G . . .    
188	C C C C C . . .    
189	T T T T T . . .    
190	G G G G G . . .    
191	A A A A A . . .    
192	A A A A A . . .    
193	G G G G G . . .    
194	C C C C C . . .    
195	T T T T T . . .    
196	C C C C C C . .    
197	A A A A A A . .    
198	G G G G G G . .    
199	G G G G G G . .    
200	G G G G G G . .    
201	G G G G G G . .    
202	A A A A A A . .    
203	A A A A A A . .    
204	T T T T T T . .    
205	T T T T T T . .    
206	A A A A A A . .    
207	T T T T T T . .    
208	T T T T T T . .    
209	T T T T T T . .    
210	G G G G G G . .    
211	T T T T T T . .    
212	T T T T T T . .    
213	T T T T T T . .    
214	C C C C C C . .    
215	T T T T T T . .    
216	A A A A A A . .    
217	T T T T T T . .    
218	A A A A A A . .    
219	G G G G G G . .    
220	T T T T T T . .    
221	A A A A A A . .    
222	C C C C C C . .    
223	T T T T T T . .    
224	A A A A A A . .    
225	T T T T T T . .    
226	A A A A A A . .    
227	A A A A A A . .    
228	A A A A A A . .    
229	G G G G G G . .    
230	A A A A A A . .    
231	T T T T T T . .    
232	G G G G G G . .    
233	C C C C C C . .    
234	A A A A A A . .    
235	C C C C C C . .    
236	C C C C C C . .    
237	A A A A A A . .    
238	T T T T T T . .    
239	C C C C C C . .    
240	T T T T T T . .    
241	T T T T T T . .    
242	C C C C C C . .    
243	A A A A A A . .    
244	A A A A A A . .    
245	A A A A A A . .    
246	G G G G G G . .    
247	T T T T T T . .    
248	T T T T T T . .    
249	A A A A A A . .    
250	G G G G G G . .    
251	G G G G G G . .    
252	A A A A A A . .    
253	T T T T T T . .    
254	T T T T T T . .    
255	T T T T T T . .    
256	A A A A A A . .    
257	A A A A A A . .    
258	G G G G G G . .    
259	T T T T T T . .    
260	G G G G G G . .    
261	C C C C C C . .    
262	T T T T T T . .    
263	T T T T T T . .    
264	T T T T T T . .    
265	T T T T T T . .    
266	T T T T T T . .    
267	G G G G G G . .    
268	T T T T T T . .    
269	T T T T T T . .    
270	G G G G G G . .    
271	T T T T T T . .    
272	T T T T T T . .    
273	A A A A A A . .    
274	A A A A A A . .    
275	A A A A A A . .    
276	T T T T T T . .    
277	G G G G G G . .    
278	G G G G G G . .    
279	A A A A A A . .    
280	C C C C C C . .    
281	T T T T T T . .    
282	G G G G G G . .    
283	G G G G G G . .    
284	G G G G G G . .    
285	A A A A A A . .    
286	G G G G G G . .    
287	A A A A A A . .    
288	C C C C C C . .    
289	C C C C C C . .    
290	T T T T T T . .    
291	T T T T T T . .    
292	T T T T T T . .    
293	C C C C C C . .    
294	A A A A A A . .    
295	G G G G G G . .    
296	A A A A A A . .    
297	T T T T T T . .    
298	T T T T T T . .    
299	T T T T T T . .    
300	G G G G G G . .    
301	G G G G G G . .    
302	T T T T T T . .    
303	G G G G G G . .    
304	G G G G G G . .    
305	C C C C C C . .    
306	T T T T T T . .    
307	G G G G G G . .    
308	G G G G G G . .    
309	A A A A A A . .    
310	T T T T T T . .    
311	T T T T T T . .    
312	T T T T T T . .    
313	A A A A A A . .    
314	A A A A A A . .    
315	C C C C C C . .    
316	A A A A A A . .    
317	C C C C C C . .    
318	T T T T T T . .    
319	G G G G G G . .    
320	A A A A A A . .    
321	C C C C C C . .    
322	C C C C C C . .    
323	C C C C C C . .    
324	A A A A A A . .    
325	T T T T T T . .    
326	A A A A A A . .    
327	T T T T T T . .    
328	G G G G G G . .    
329	A A A A A A . .    
330	A A A A A A . .    
331	T T T T T T . .    
332	C C C C C C . .    
333	T T T T T T . .    
334	G G G G G G . .    
335	A A A A A A . .    
336	T T T T T T . .    
337	G G G G G G . .    
338	C C C C C C . .    
339	C C C C C C . .    
340	C C C C C C . .    
341	A A A A A A . .    
342	C C C C C C . .    
343	A A A A A A . .    
344	G G G G G G . .    
345	A A A A A A . .    
346	T T T T T T . .    
347	A A A A A A . .    
348	T C C C C C . .    
349	A A A A A A . .    
350	A A A A A A . .    
351	G G G G G G . .    
352	T T T T T T . .    
353	C C C C C C . .    
354	A A A A A A . .    
355	C C C C C C . .    
356	A A A A A A . .    
357	A A A A A A . .    
358	C C C C C C . .    
359	A A A A A A . .    
360	A A A A A A . .    
361	A A A A A A . .    
362	G G G G G G . .    
363	T T T T T T . .    
364	A A A A A A . .    
365	C C C C C C . .    
366	A A A A A A . .    
367	C C C C C C . .    
368	A A A A A A . .    
369	A A A A A A . .    
370	T C C C C C . .    
371	T T T T T T . .    
372	G G G G G G . .    
373	A A A A A A . .    
374	A A A A A A . .    
375	G G G G G G . .    
376	A A A A A A . .    
377	G G G G G G . .    
378	A A A A A A . .    
379	C C C C C C . .    
380	A A A A A A . .    
381	T T T T T T . .    
382	T T T T T T . .    
383	G G G G G G . .    
384	A A A A A A . .    
385	G G G G G G . .    
386	G G G G G G . .    
387	T T T T T T . .    
388	A A A A A A . .    
389	A A A A A A . .    
390	A A A A A A . .    
391	C C C C C C . .    
392	A A A A A A . .    
393	G G G G G G . .    
394	G G G G G G . .    
395	T T T T T T . .    
396	A A A A A A . .    
397	C C C C C C . .    
398	G G G G G G . .    
399	C C C C C C . .    
400	A A A A A A . .    
401	T T T T T T . .    
402	A A A A A A . .    
403	T T T T T T . .    
404	A A A A A A . .    
405	C C C C C C . .    
406	T T T T T T . .    
407	G G G G G G . .    
408	T T T T T T . .    
409	C C C C C C . .    
410	T T T T T T . .    
411	T T T T T T . .    
412	A A A A A A . .    
413	G G G G G G . .    
414	C C C C C C . .    
415	A A A A A A . .    
416	A A A A A A . .    
417	C C C C C C . .    
418	T T T T T T . .    
419	A A A A A A . .    
420	T T T T T T . .    
421	A A A A A A . .    
422	A A A A A A . .    
423	G G G G G G . .    
424	A A A A A A . .    
425	A A A A A A . .    
426	A A A A A A . .    
427	G G A A G G . .    
428	A A A A A A . .    
429	A A A A A A . .    
430	T T T T T T . .    
431	A A A A A A . .    
432	C C C C C C . .    
433	T T T T T T . .    
434	A A A A A A . .    
435	A A A A A A . .    
436	C C C C C C . .    
437	A A A A A A . .    
438	G G G G G G . .    
439	C C C C C C . .    
440	T T T T T T . .    
441	G G G G G G . .    
442	T T T T T T . .    
443	T T T T T T . .    
444	T T T T T T . .    
445	C C C C C C . .    
446	C C C C C C . .    
447	A A A A A A . .    
448	T T T T T T . .    
449	T T T T T T . .    
450	G G G G G G . .    
451	A A A A A A . .    
452	A A A A A A . .    
453	A A A A A A . .    
454	T T T T T T . .    
455	A A A A A A . .    
456	A A A A A A . .    
457	G G G G G G . .    
458	G G G G G G . .    
459	C C C C C C . .    
460	A A A A A A . .    
461	A A A A A A . .    
462	A A A A A A . .    
463	T T T T T T . .    
464	A A A A A A . .    
465	C C C C C C . .    
466	A A A A A A . .    
467	G G G G G G . .    
468	T T T T T T . .    
469	A A A A A A . .    
470	T T T T T T . .    
471	T T T T T T . .    
472	G G G G G G . .    
473	T T T T T T . .    
474	A A A A A A . .    
475	A A A A A A . .    
476	A A A A A A . .    
477	C C C C C C . .    
478	T T T T T T . .    
479	G G G G G G . .    
480	T T T T T T . .    
481	C C C C C C . .    
482	A A A A A A . .    
483	A A A A A A . .    
484	A A A A A A . .    
485	A A A A A A . .    
486	C C C C C C . .    
487	C C C C C C . .    
488	A A A A A A . .    
489	T T T T T T . .    
490	T T T T T T . .    
491	T T T T T T . .    
492	G G G G G G . .    
493	C C C C C C . .    
494	C C C C C C . .    
495	G G G G G G . .    
496	G G G G G G . .    
497	T T T T T T . .    
498	T T T T T T . .    
499	T T T T T T . .    
500	T T T T T T . .    
501	C C C C C C . .    
502	A A A A A A . .    
503	A A A A A A . .    
504	A A A A A A . .    
505	A A A A A A . .    
506	T T T T T T . .    
507	T T T T T T . .    
508	A A A A A A . .    
509	A A A A A A . .    
510	A A A A A A . .    
511	G G G G G G . .    
512	T T T T T T . .    
513	G G G G G G . .    
514	A A A A A A . .    
515	C C C C C C . .    
516	T T T T T T . .    
517	T T T T T T . .    
518	T T T T T T . .    
519	G G G G G G . .    
520	A A A A A A . .    
521	A A A A A A A .    
522	T T T T T T T .    
523	T T T T T T T .    
524	T T T T T T T .    
525	A A A A A A A .    
526	A A A A A A A .    
527	A A A A A A A .    
528	T T T T T T T .    
529	T T T T T T T .    
530	T T T T T T T .    
531	A A A A A A A .    
532	G G G G G G G .    
533	A A A A A A A .    
534	G G G G G G G .    
535	G G G G G G G .    
536	A A A A A A A .    
537	A A A A A A A .    
538	C C C C C C C .    
539	A A A A A A A .    
540	G G G G G G G .    
541	A A A A A A A .    
542	G G G G G G G .    
543	G G G G G G G .    
544	A A A A A A A .    
545	A A A A A A A .    
546	A A A A A A A .    
547	G G G G G G G .    
548	A A A A A A A .    
549	A A A A A A A .    
550	T T T T T T T .    
551	A A A A A A A .    
552	C C C C C C C .    
553	A A A A A A A .    
554	A A A A A A A .    
555	A A A A A A A .    
556	T T T T T T T .    
557	A A A A A A A .    
558	A A A A A A A .    
559	T T T T T T T .    
560	A A A A A A A .    
561	A A A A A A A .    
562	A A A A A A A .    
563	G G G G G G G .    
564	T T T T T T T .    
565	C C C C C C C .    
566	C C C C C C C .    
567	A A A A A A A .    
568	G G G G G G G .    
569	T T T T T T T .    
570	A A A A A A A .    
571	A A A A A A A .    
572	C C C C C C C .    
573	A A A A A A A .    
574	A A A A A A A .    
575	T T T T T T T .    
576	A A A A A A A .    
577	C C C C C C C .    
578	A A A A A A A .    
579	T T T T T T T .    
580	T T T T T T T .    
581	C C C C C C C .    
582	T T T T T T T .    
583	A A A A A A A .    
584	C C C C C C C .    
585	T T T T T T T .    
586	A A A A A A A .    
587	G G G G G G G .    
588	T T T T T T T .    
589	G G G G G G G .    
590	C C C C C C C .    
591	A A A A A A A .    
592	T T T T T T T .    
593	T T T T T T T .    
594	G G G G G G G .    
595	T T T T T T T .    
596	G G G G G G G .    
597	G G G G G G G .    
598	A A A A A A A .    
599	T T T T T T T .    
600	T T T T T T T .    
601	A A A A A A A .    
602	C C C C C C C .    
603	T T T T T T T .    
604	G G G G G G G .    
605	C C C C C C C .    
606	T T T T T T T .    
607	T T T T T T T .    
608	C C C C C C C .    
609	G G G G G G G .    
610	T T T T T T T .    
611	A A A A A A A .    
612	A A A A A A A .    
613	T T T T T T T .    
614	G G G G G G G .    
615	A A A A A A A .    
616	A A A A A A A .    
617	A A A A A A A .    
618	A A A A A A A .    
619	C C C C C C C .    
620	T T T T T T T .    
621	G G G G G G G .    
622	A A A A A A A .    
623	C C C C C C C .    
624	C C C C C C C .    
625	T T T T T T T .    
626	T T T T T T T .    
627	G G G G G G G .    
628	G G G G G G G .    
629	A A A A A A A .    
630	A A A A A A A .    
631	A A A A A A A .    
632	T T T T T T T .    
633	T T T T T T T .    
634	C C C C C C C .    
635	A A A A A A A .    
636	T T T T T T T .    
637	G G G G G G G .    
638	A A A A A A A .    
639	A A A A A A A .    
640	A A A A A A A .    
641	G G G G G G G .    
642	C C C C C C C .    
643	T T T T T T T .    
644	A A A A A A A .    
645	A A A A A A A .    
646	T T T T T T T .    
647	A A A A A A A .    
648	C C C C C C C .    
649	T T T T T T T .    
650	T T T T T T T .    
651	T C C C C C C .    
652	. A A A A A A .    
653	. A A A A A A .    
654	. T T T T T T .    
655	. T T T T T T .    
656	. A A A A A A .    
657	. T T T T T T .    
658	. A A A A A A .    
659	. T T T T T T .    
660	. G G G G G G .    
661	. T T T T T T .    
662	. A A A A A A .    
663	. T T T T T T .    
664	. A A A A A A .    
665	. C C C C C C .    
666	. A A A A A A .    
667	. A A A A A A .    
668	. T T T T T T .    
669	. A A A A A A .    
670	. T T T T T T .    
671	. A A A A A A .    
672	. A A A A A A .    
673	. T T T T T T .    
674	. C C C C C C .    
675	. A A A A A A .    
676	. T T T T T T .    
677	. G A A G G A .  3	 transition  
678	. G T T G G T .  3	 transversion  
679	. - A A - - A .  3	 indel  
680	. - A A - - A .  3	 indel  
681	. T T T T T T .    
682	. C . C C C C .    
683	. A . A A A A .    
684	. A . A A A A .    
685	. T . T T T T .    
686	. T . T T T T .    
687	. G . G G G G .    
688	. C . C C C C .    
689	. A . A A A A .    
690	. T . T T T T .    
691	. T . T T T T .    
692	. A . A A A A .    
693	. G . G G G G .    
694	. T . T T T T .    
695	. C . C C C C .    
696	. T . T T T T .    
697	. T . T T T T .    
698	. G . G G G G .    
699	. G . G G G G .    
700	. T . T T T T .    
701	. T . T T T T .    
702	. T . T T T T .    
703	. G . G G G G .    
704	. A . A A A A .    
705	. C . C C C C .    
706	. C . C C C C .    
707	. C . C C C C .    
708	. T . T T T T .    
709	. . . C C C C .    
710	. . . T T T T .    
711	. . . G G G G .    
712	. . . T T T T .    
713	. . . G G G G .    
714	. . . A A A A .    
715	. . . C C C C .    
716	. . . T T T T .    
717	. . . T T T T .    
718	. . . T T T T .    
719	. . . G G G G .    
720	. . . A A A A .    
721	. . . T T T T .    
722	. . . G G G G .    
723	. . . C C C C .    
724	. . . T T T T .    
725	. . . G G G G .    
726	. . . T T T T .    
727	. . . T T T T .    
728	. . . T T T T .    
729	. . . T T T T .    
730	. . . T T T T .    
731	. . . A A A A .    
732	. . . T T T T .    
733	. . . T T T T .    
734	. . . T T T T .    
735	. . . A A A A .    
736	. . . G G G G .    
737	. . . C C C C .    
738	. . . T T T T .    
739	. . . A A A A .    
740	. . . A A A A .    
741	. . . A A A A .    
742	. . . G G G G .    
743	. . . G G G G .    
744	. . . A A A A .    
745	. . . A A A A .    
746	. . . G G G G .    
747	. . . . T T T .    
748	. . . . T T T .    
749	. . . . A A A .    
750	. . . . G G G .    
751	. . . . T T T .    
752	. . . . G G G .    
753	. . . . T T T .    
754	. . . . C C C .    
755	. . . . A A A .    
756	. . . . G G G .    
757	. . . . A A A .    
758	. . . . G G G .    
759	. . . . A A A .    
760	. . . . A A A .    
761	. . . . A A A .    
762	. . . . G G G G    
763	. . . . T T T T    
764	. . . . A A A A    
765	. . . . C C C C    
766	. . . . A A A A    
767	. . . . T T T T    
768	. . . . A A A A    
769	. . . . C C C C    
770	. . . . A A A A    
771	. . . . G G G G    
772	. . . . T T T T    
773	. . . . A A A A    
774	. . . . T T T T    
775	. . . . G G G G    
776	. . . . C C C C    
777	. . . . A A A A    
778	. . . . A A A A    
779	. . . . A A A A    
780	. . . . T T T T    
781	. . . . G G G G    
782	. . . . A A A A    
783	. . . . A A A A    
784	. . . . T T T T    
785	. . . . T T T T    
786	. . . . C C C C    
787	. . . . A A A A    
788	. . . . T T T T    
789	. . . . T T T T    
790	. . . . T T T T    
791	. . . . T T T T    
792	. . . . A A A A    
793	. . . . C C C C    
794	. . . . T T T T    
795	. . . . G G G G    
796	. . . . A A A A    
797	. . . . G G G G    
798	. . . . A A A A    
799	. . . . T T T T    
800	. . . . A A A A    
801	. . . . A A A A    
802	. . . . T T T T    
803	. . . . T T T T    
804	. . . . A A A A    
805	. . . . G G G G    
806	. . . . A A A A    
807	. . . . A A A A    
808	. . . . T T T T    
809	. . . . A A A A    
810	. . . . C C C C    
811	. . . . T T T T    
812	. . . . G G G G    
813	. . . . T T T T    
814	. . . . A A A A    
815	. . . . A A A A    
816	. . . . C C C C    
817	. . . . T T T T    
818	. . . . A A A A    
819	. . . . C C C T    
820	. . . . A A A A    
821	. . . . T T T T    
822	. . . . T T T T    
823	. . . . A A A A    
824	. . . . G G G G    
825	. . . . A A A A    
826	. . . . C C C C    
827	. . . . T T T T    
828	. . . . A A A A    
829	. . . . T T T T    
830	. . . . A A A A    
831	. . . . A A A A    
832	. . . . C C C C    
833	. . . . A A A A    
834	. . . . T T T T    
835	. . . . T T T T    
836	. . . . T T T T    
837	. . . . T T T T    
838	. . . . A A A A    
839	. . . . C C C C    
840	. . . . A A A A    
841	. . . . A A A A    
842	. . . . C C C C    
843	. . . . A A A A    
844	. . . . A A A A    
845	. . . . T T T T    
846	. . . . G G G G    
847	. . . . T T T T    
848	. . . . T T T T    
849	. . . . A A A A    
850	. . . . A A A A    
851	. . . . A A A A    
852	. . . . T T T T    
853	. . . . A A A A    
854	. . . . T T T T    
855	. . . . A A A A    
856	. . . . A A A A    
857	. . . . T T T T    
858	. . . . C C C C    
859	. . . . T T T T    
860	. . . . A A A A    
861	. . . . A A A A    
862	. . . . A A A A    
863	. . . . A A A A    
864	. . . . A A A A    
865	. . . . C C C C    
866	. . . . . T T T    
867	. . . . . C C C    
868	. . . . . C C C    
869	. . . . . T T T    
870	. . . . . A A A    
871	. . . . . T T T    
872	. . . . . C C C    
873	. . . . . A A A    
874	. . . . . G G G    
875	. . . . . A A C    
876	. . . . . A A A    
877	. . . . . A A A    
878	. . . . . T T T    
879	. . . . . T T T    
880	. . . . . G G G    
881	. . . . . C C C    
882	. . . . . A A A    
883	. . . . . C C C    
884	. . . . . A A A    
885	. . . . . C C C    
886	. . . . . T T T    
887	. . . . . G G G    
888	. . . . . A A A    
889	. . . . . A A A    
890	. . . . . A A A    
891	. . . . . G G G    
892	. . . . . A A A    
893	. . . . . A A A    
894	. . . . . G G G    
895	. . . . . T T T    
896	. . . . . G G G    
897	. . . . . T T T    
898	. . . . . G G G    
899	. . . . . T T T    
900	. . . . . T T T    
901	. . . . . T T T    
902	. . . . . C C C    
903	. . . . . A A A    
904	. . . . . T T T    
905	. . . . . C C C    
906	. . . . . A A A    
907	. . . . . A A A    
908	. . . . . T T T    
909	. . . . . T T T    
910	. . . . . G G G    
911	. . . . . C C C    
912	. . . . . A A A    
913	. . . . . A A A    
914	. . . . . C C C    
915	. . . . . A A A    
916	. . . . . A A A    
917	. . . . . A A A    
918	. . . . . C C C    
919	. . . . . A A A    
920	. . . . . C C C    
921	. . . . . A A A    
922	. . . . . A A A    
923	. . . . . T T T    
924	. . . . . T T T    
925	. . . . . T T T    
926	. . . . . G G G    
927	. . . . . T T T    
928	. . . . . T T T    
929	. . . . . G G G    
930	. . . . . C C C    
931	. . . . . A A A    
932	. . . . . A A A    
933	. . . . . A A A    
934	. . . . . C C C    
935	. . . . . A A A    
936	. . . . . C C C    
937	. . . . . A A A    
938	. . . . . T T T    
939	. . . . . T T T    
940	. . . . . A A A    
941	. . . . . C C C    
942	. . . . . A A A    
943	. . . . . G G G    
944	. . . . . A A A    
945	. . . . . A A A    
946	. . . . . A A A    
947	. . . . . C C C    
948	. . . . . A A A    
949	. . . . . T T T    
950	. . . . . T T T    
951	. . . . . A A A    
952	. . . . . A A A    
953	. . . . . G G G    
954	. . . . . T T T    
955	. . . . . G G G    
956	. . . . . T T T    
957	. . . . . T T T    
958	. . . . . G G G    
959	. . . . . A A A    
960	. . . . . A A A    
961	. . . . . A A A    
962	. . . . . T T T    
963	. . . . . G G G    
964	. . . . . T T T    
965	. . . . . A A A    
966	. . . . . T T T    
967	. . . . . T T T    
968	. . . . . C C C    
969	. . . . . A A A    
970	. . . . . T T T    
971	. . . . . T T T    
972	. . . . . T T T    
973	. . . . . T T T    
974	. . . . . G G G    
975	. . . . . T T T    
976	. . . . . C C C    
977	. . . . . A A A    
978	. . . . . C C C    
979	. . . . . T T T    
980	. . . . . A A A    
981	. . . . . A A A    
982	. . . . . G G G    
983	. . . . . G G G    
984	. . . . . T T T    
985	. . . . . T T T    
986	. . . . . A A A    
987	. . . . . G G G    
988	. . . . . G G G    
989	. . . . . C C C    
990	. . . . . A A A    
991	. . . . . T T T    
992	. . . . . T T T    
993	. . . . . C C C    
994	. . . . . A A A    
995	. . . . . C C C    
996	. . . . . T T T    
997	. . . . . C C C    
998	. . . . . A A A    
999	. . . . . G G G    
1000	. . . . . T T T    
1001	. . . . . A A A    
1002	. . . . . T T T    
1003	. . . . . T T T    
1004	. . . . . T T T    
1005	. . . . . T T T    
1006	. . . . . G G G    
1007	. . . . . G G G    
1008	. . . . . A A A    
1009	. . . . . C C C    
1010	. . . . . T T T    
1011	. . . . . G G G    
1012	. . . . . T T T    
1013	. . . . . G G G    
1014	. . . . . T T C    
1015	. . . . . C C C    
1016	. . . . . A A A    
1017	. . . . . T T T    
1018	. . . . . G G G    
1019	. . . . . C C C    
1020	. . . . . C C C    
1021	. . . . . A A A    
1022	. . . . . G G G    
1023	. . . . . G G G    
1024	. . . . . C C C    
1025	. . . . . G G G    
1026	. . . . . T T T    
1027	. . . . . G G G    
1028	. . . . . A A A    
1029	. . . . . C C C    
1030	. . . . . A A A    
1031	. . . . . G G G    
1032	. . . . . A A A    
1033	. . . . . C C C    
1034	. . . . . C C C    
1035	. . . . . A A A    
1036	. . . . . A A A    
1037	. . . . . T T T    
1038	. . . . . A A A    
1039	. . . . . C C C    
1040	. . . . . C C C    
1041	. . . . . C A A    
1042	. . . . . . C C    
1043	. . . . . . A A    
1044	. . . . . . A A    
1045	. . . . . . C C    
1046	. . . . . . A A    
1047	. . . . . . A A    
1048	. . . . . . C C    
1049	. . . . . . T T    
1050	. . . . . . A A    
1051	. . . . . . G G    
1052	. . . . . . A A    
1053	. . . . . . C C    
1054	. . . . . . T T    
1055	. . . . . . T T    
1056	. . . . . . C C    
1057	. . . . . . A A    
1058	. . . . . . T T    
1059	. . . . . . T T    
1060	. . . . . . T T    
1061	. . . . . . A A    
1062	. . . . . . C C    
1063	. . . . . . C C    
1064	. . . . . . G G    
1065	. . . . . . A A    
1066	. . . . . . T T    
1067	. . . . . . G G    
1068	. . . . . . A A    
1069	. . . . . . A A    
1070	. . . . . . A A    
1071	. . . . . . T T    
1072	. . . . . . T T    
1073	. . . . . . T T    
1074	. . . . . . A A    
1075	. . . . . . G G    
1076	. . . . . . T T    
1077	. . . . . . C C    
1078	. . . . . . A A    
1079	. . . . . . T T    
1080	. . . . . . A A    
1081	. . . . . . T T    
1082	. . . . . . A A    
1083	. . . . . . A A    
1084	. . . . . . C C    
1085	. . . . . . A A    
1086	. . . . . . C C    
1087	. . . . . . T T    
1088	. . . . . . A A    
1089	. . . . . . T T    
1090	. . . . . . T T    
1091	. . . . . . G G    
1092	. . . . . . T T    
1093	. . . . . . T T    
1094	. . . . . . G G    
1095	. . . . . . T T    
1096	. . . . . . C C    
1097	. . . . . . C C    
1098	. . . . . . T T    
1099	. . . . . . G G    
1100	. . . . . . G G    
1101	. . . . . . A A    
1102	. . . . . . A A    
1103	. . . . . . A A    
1104	. . . . . . A A    
1105	. . . . . . A A    
1106	. . . . . . G G    
1107	. . . . . . G G    
1108	. . . . . . A A    
1109	. . . . . . G G    
1110	. . . . . . C C    
1111	. . . . . . T T    
1112	. . . . . . C C    
1113	. . . . . . A A    
1114	. . . . . . A A    
1115	. . . . . . C C    
1116	. . . . . . T T    
1117	. . . . . . A A    
1118	. . . . . . C C    
1119	. . . . . . A A    
1120	. . . . . . A A    
1121	. . . . . . G G    
1122	. . . . . . T T    
1123	. . . . . . C C    
1124	. . . . . . T T    
1125	. . . . . . T T    
1126	. . . . . . G G    
1127	. . . . . . C C    
1128	. . . . . . C C    
1129	. . . . . . C C    
1130	. . . . . . T T    
1131	. . . . . . G G    
1132	. . . . . . A A    
1133	. . . . . . A A    
1134	. . . . . . A A    
1135	. . . . . . A A    
1136	. . . . . . A A    
1137	. . . . . . A A    
1138	. . . . . . T T    
1139	. . . . . . G G    
1140	. . . . . . C C    
1141	. . . . . . T T    
1142	. . . . . . T T    
1143	. . . . . . C C    
1144	. . . . . . T T    
1145	. . . . . . A A    
1146	. . . . . . T T    
1147	. . . . . . A A    
1148	. . . . . . A A    
1149	. . . . . . A A    
1150	. . . . . . A A    
1151	. . . . . . T T    
1152	. . . . . . T T    
1153	. . . . . . C C    
1154	. . . . . . T T    
1155	. . . . . . G G    
1156	. . . . . . T T    
1157	. . . . . . A A    
1158	. . . . . . A A    
1159	. . . . . . T T    
1160	. . . . . . A A    
1161	. . . . . . G G    
1162	. . . . . . T T    
1163	. . . . . . A A    
1164	. . . . . . A A    
1165	. . . . . . A A    
1166	. . . . . . A A    
1167	. . . . . . A A    
1168	. . . . . . A A    
1169	. . . . . . A A    
1170	. . . . . . G G    
1171	. . . . . . T T    
1172	. . . . . . T T    
1173	. . . . . . A A    
1174	. . . . . . T T    
1175	. . . . . . C A    
1176	. . . . . . . A    
1177	. . . . . . . T    
1178	. . . . . . . A    
1179	. . . . . . . A    
1180	. . . . . . . T    
1181	. . . . . . . T    
1182	. . . . . . . G    
1183	. . . . . . . A    
1184	. . . . . . . T    
1185	. . . . . . . T    
1186	. . . . . . . G    
1187	. . . . . . . G    
1188	. . . . . . . A    
1189	. . . . . . . T    
1190	. . . . . . . T    
1191	. . . . . . . T    
1192	. . . . . . . A    
1193	. . . . . . . T    
1194	. . . . . . . A    
1195	. . . . . . . T    
1196	. . . . . . . A    
1197	. . . . . . . G    
1198	. . . . . . . T    
1199	. . . . . . . G    
1200	. . . . . . . C    
1201	. . . . . . . T    
1202	. . . . . . . T    
1203	. . . . . . . T    
1204	. . . . . . . T    
1205	. . . . . . . C    
1206	. . . . . . . T    
1207	. . . . . . . A    
1208	. . . . . . . C    
1209	. . . . . . . C    
1210	. . . . . . . A    
1211	. . . . . . . A    
1212	. . . . . . . C    
1213	. . . . . . . T    
1214	. . . . . . . G    
1215	. . . . . . . A    
1216	. . . . . . . G
```
